# Extracellular Vesicles From Notch Activated Cardiac Mesenchymal Stem Cells Promote Myocyte Proliferation and Neovasculogenesis

**DOI:** 10.3389/fcell.2020.00011

**Published:** 2020-02-21

**Authors:** Wanling Xuan, Mahmood Khan, Muhammad Ashraf

**Affiliations:** ^1^Vascular Biology Center, Department of Medicine, Medical College of Georgia, Augusta University, Augusta, GA, United States; ^2^Department of Emergency Medicine, Wexner Medical Center, The Ohio State University, Columbus, OH, United States

**Keywords:** cardiac mesenchymal stem cells, extracellular vesicles, Notch1, angiogenesis, proliferation, myocardial infarction

## Abstract

Cardiac mesenchymal stem cells (C-MSCs) are a novel mesenchymal stem cell (MSC) subpopulation derived from cardiac tissue, which are reported to be responsible for cardiac regeneration. Notch signaling is believed to aid in cardiac repair following myocardial injury. In this study, we have investigated the role of extracellular vesicles (EVs) from Notch1 engineered C-MSCs on angiogenesis and cardiomyocyte (CM) proliferation in ischemic myocardium. C-MSCs were isolated from Notch1^flox^ mice (C-MSC^Notch1 FF^). Notch1 gene deletion was accomplished by adenoviral vector-mediated Cre recombination, and Notch1 overexpression was achieved by overexpression of Notch1 intracellular domain (N1ICD). EVs were isolated by using the size exclusion column method. Proteomic composition of EV was carried out by mass spectrometry. A mouse myocardial infarction (MI) model was generated by permanent left anterior descending (LAD) coronary artery ligation. Intramyocardial transplantation of Notch1 knockout C-MSCs (C-MSCs^Notch1 KO^) did not have any effect on cardiac function and scar size. On the other hand, transplantation of N1ICD-overexpressing C-MSCs (C-MSCs^N1ICD^) showed significant improvement in cardiac function and attenuation of fibrosis as compared to the control (PBS) group and non-modified C-MSC groups. C-MSCs^N1ICD^ differentiated into smooth muscle cells and formed new vessels. Proteomics profiling identified several proteins, such as lysyl oxidase homolog-2 and biglycan, as highly enriched proteins in EV-C-MSCs^N1ICD^. Go term analysis indicated that EV-C-MSCs^N1ICD^ were enriched with bioactive factors, potent pro-repair proteins responsible for cell migration and proliferation. EV-C-MSCs^*Notch1FF*^ and EV-C-MSCs^N1ICD^ were strongly proangiogenic under both *in vitro* and *in vivo* conditions. EV-C-MSCs^N1ICD^ caused dense tube formation *in vitro* and increased neovasculogenesis in the peri-infarct area *in vivo*. Furthermore, EV-C-MSCs^N1ICD^ attenuated endothelial cell (EC) and CM apoptosis under oxidative stress and ischemic injury. Similarly, EV-C-MSC^Notch1 FF^ and EV-C-MSC^N1ICD^ treatment improved cardiac function and decreased fibrosis in mice post-MI. EV-C-MSCs^N1ICD^ were very effective in improving cardiac function and decreasing fibrosis. Notch1 signaling is a strong stimulus for cardiac regeneration by C-MSCs. EVs secreted by Notch1-overexpressing C-MSCs were highly effective in preventing cell death, promoting angiogenesis and CM proliferation, and restoring cardiac function post-MI. Overall, these results suggest that Notch1 overexpression may further enhance the effectiveness of EVs secreted by C-MSCs in cell-free therapy.

## Introduction

Cardiac mesenchymal stem cells (C-MSCs) are a novel mesenchymal stem cell (MSC) subpopulation arising from cardiac tissue, which are predominantly Sca-1^+^ cells and express mesenchymal surface antigens. Compared with bone marrow-derived MSCs (BM-MSCs), C-MSCs expressed GATA4 (an early cardiac transcription factor marker) (Chen et al., 2012). C-MSCs have the advantage of being preconditioned by the cardiac micro-environment and epigenetic profile, which showed enhanced levels of histone acetylation at the promoter regions of the cardiac-specific genes (Wang et al., 2013). Although these stem cells are capable of transforming into new cardiac cells, their contribution toward functional improvement of the heart under ischemic conditions remains controversial.

Cardiac mesenchymal stem cells play an important role in angiogenesis of the ischemic heart. A lineage tracing study demonstrated that cardiac-resident Sca-1^+^ cells abundantly contributed to the cardiac vasculature in mice during physiological growth and during cardiac remodeling after myocardial infarction (MI) (Vagnozzi et al., 2018). Thus, C-MSCs might serve as a potential candidate for cell therapy. However, differentiation of C-MSCs is believed to depend on close cell-to-cell interaction via Notch1 signaling as trypsinization causes a loss of cell–cell contact and impairs C-MSC differentiation during *in vivo* transplantation (Li et al., 2006).

Notch signaling is involved in mammalian cardiogenesis, including cell fate decisions, differentiation and proliferation, formation of heart tissues, and angiogenesis (del Monte et al., 2011; MacGrogan et al., 2014; Zhou and Liu, 2014). Key signaling pathways responsible for cardiac morphogenesis become transiently reactivated in the damaged heart. Multiple studies reported that Notch signaling was reactivated during myocardial injury and initiated cardiac repair following myocardial injury (Li et al., 2010; Nistri et al., 2017). Notch signaling determines both the extent of myocardial damage and pathological left ventricular remodeling involving regeneration, cardiomyocyte (CM) survival, fibrotic response, and angiogenesis (Rizzo et al., 2014). For instance, activation of Notch1 in neonatal rat CMs and intact mouse myocardium elevated phospho-Akt^S473^ levels as well as proliferation of myocytes in the infarcted heart (Gude et al., 2008). The cardioprotective effect of Notch1 against ischemic damage was reported to be mediated by AMPK signaling via an interaction with upstream liver kinase beta 1 (LKB1) (Yang et al., 2016). Moreover, both systemic and BM-MSC-specific ablation of Notch1 led to impaired cardiac repair following MI (Li et al., 2011). However, the role of Notch1 signaling in C-MSCs remains unclear.

A previous study reported that overexpression of Notch1 intracellular domain (N1ICD), the active form of Notch1, promoted cardiosphere derived cells (CDCs) toward vascular smooth muscle cell (VSMC) differentiation both *in vitro* and *in vivo* (Chen et al., 2012). In this regard, Notch1 activation in C-MSCs might potentially stimulate vascular repair by angiogenesis. It has been demonstrated that C-MSC administration improved cardiac function in animal models of heart failure (Moore et al., 2017). Nevertheless, whether Notch1 overexpression could further enhance the regenerative capability of C-MSCs and improve cardiac function remains unclear and requires further investigation. More recently, extracellular vesicles (EVs) secreted by stem cells have been reported as final effectors of protection against ischemic injury. These EVs carry miRNAs and proteins which facilitate cell–cell communication in addition to other cellular effects (Mathieu et al., 2019). Bioactive molecules in EVs secreted by C-MSCs presumably contributed to the afforded benefits (Wysoczynski et al., 2019). Therefore, the present study was designed to investigate whether Notch1 overexpression in C-MSCs could render their EVs more effective in cardiac repair following MI.

## Materials and Methods

### C-MSC Isolation and Culture

Mouse C-MSCs were obtained from Dr. Yaoliang Tang at Augusta University, which were isolated from the hearts of 2- to 3-month-old Notch1^flox^ mice (The Jackson Laboratory, stock number: 006951) according to the procedure as previously described (Ju et al., 2018). The isolated cells were purified using a mouse hematopoietic lineage depletion cocktail kit (Stemcell Technologies) and Sca-1 magnetic beads (MiltenyiBiotec Inc.) with magnetic activated cell sorting (MACS). These cells expressed the mesenchymal cell surface makers CD105, CD44, and CD140 by flow cytometric analyses (Ju et al., 2018). Cells were cultured in high-glucose DMEM medium supplemented with 10% fetal bovine serum (FBS), 200 mM L-glutamine, 55 nM β-mercaptoethanol, and 1% MEM non-essential amino acid. Before EV collection, culture medium was switched to medium supplemented with exosome-deleted FBS (Gibco) for 48 h. C-MSCs isolated from Notch1^flox^ mice were designated as C-MSCs^Notch1^ ^FF^.

### Generation of Notch1 Knockout C-MSCs (C-MSCs^Notch1 KO^) and N1ICD Overexpression in C-MSCs (C-MSCs^N1ICD^)

Notch1 knockout C-MSCs were generated by deletion of Notch1 genes in C-MSCs isolated from Notch1^flox^ mice via adenoviral vector–mediated Cre recombination. N1ICD-overexpressing C-MSCs were generated via adenoviral vector-mediated transient overexpression of N1ICD. N1ICD recombinant adenovirus was generated as described in a previous study (Chen et al., 2012). The expression of N1ICD in C-MSCs was determined by western blot.

### Mouse Aortic Endothelial Cell Isolation and Culture

Primary mouse aortic endothelial cells (ECs) were isolated as described previously (Wang et al., 2016). Briefly, the mouse thoracic aorta was quickly removed using micro-dissection forceps and gently flushed with ice-cold PBS to remove the blood. Then the aorta was cut into 1 mm rings. These aortic segments were cultured on matrix in EC growth medium for EC sprouting. After 2–3 days, the proliferating ECs were harvested and passaged. The cells were characterized by CD31 staining.

### Human EC Culture

Human cardiac microvascular ECs (CMVECs, CC-7030) and human aortic ECs (HAECs, CC-2535) were obtained from Lonza Company. Cells were maintained in EC growth medium V-2 (213-500, CELL APPLICATIONS, Inc.). Cells at passage 2–6 were used for experiments.

### Isolation of EVs

Extracellular vesicles were isolated using the size exclusion column method. Briefly, conditioned media were collected, and EVs were isolated by centrifugation at 3000 r/min for 30 min to remove cells and debris, followed by filtration through a 0.22 μm filter to remove the remaining debris. Then the medium was further concentrated using Amicon Ultra-15 100 kDa centrifugal filter units (Millipore). Isolation of EVs in the concentrated medium was carried out through qEV size exclusion columns (Izon Science). EV fractions were collected and concentrated by an Amicon Ultra-4 10 kDa centrifugal filter (Millipore). The purified EVs were stored at -80°C and subsequently characterized by particle size, electron microscopy, and proteomic profile.

### Concentration and Particle Size Measurement With Tunable Resistive Pulse Sensing

Particle size and concentration were analyzed using the tunable resistive pulse sensing (TRPS) technique with a qNano instrument (Izon Science) as described in a previous study (Vogel et al., 2016). Briefly, the number of particles was counted (at least 600–1000 events) at 20 mbar pressure. Beads of CPC200 (200 nm) were used for calibration. Data were analyzed using Izon Control Suite software.

### Transmission Electron Microscopy

Tissue samples were processed for transmission electron microscopy (TEM) by the Electron Microscopy and Histology Core Laboratory at Augusta University. Briefly, EV suspension was fixed with an equal volume of 8% paraformaldehyde (PFA) to preserve ultrastructure. Ten microliters of suspended/fixed exosomes was applied to a carbon/formvar-coated 200 mesh copper grid and allowed to stand for 30–60 s. The excess was absorbed by Whatman filter paper. Ten microliters of 2% aqueous uranyl acetate was added and treated for 30 s. Grids were allowed to air-dry before being examined in a JEM 1230 transmission electron microscope (JEOL USA Inc., Peabody, MA, United States) at 110 kV and imaged with an UltraScan 4000 CCD camera and First Light Digital Camera Controller (Gatan Inc., Pleasanton, CA, United States).

### EV Internalization

Purified EVs were labeled with PKH26 (Sigma–Aldrich), according to the manufacturer’s protocol. Briefly, 300 μL of EVs was suspended into 100 μL of Diluent C, which was mixed with 1.4 μL of PKH26 dye. The labeling reaction was stopped by adding an equal volume of exosome-free FBS. Exosome Spin Columns (Thermo Fisher Scientific) were used to remove unincorporated PKH26. The CMVECs were incubated with labeled EVs for 3 h. After incubation, cells were stained with WGA conjugated with Alexa Fluor 488 (Thermo Fisher Scientific). Cells were fixed with 2% formaldehyde for 5 min and mounted with DAPI mounting media (Southern Biotech). Images were taken with a fluorescent microscope (Olympus, Japan).

### Tube Formation Assay

HAECs (1 × 10^5^ cells/well) were seeded on Matrigel (Corning) in a 24-well plate and treated with or without 1 μg EVs from different groups of C-MSCs in EGM-2V basal medium (Lonza). Complete EGM-2V medium was used as positive control. After 16 h, cells in Matrigel were stained with Calcein AM, and images were taken by fluorescent microscope. Tube formation was analyzed by Wimasis Image Analysis Platform.

### Induction of Myocardial Infarction

Animal experiments were carried out according to experimental protocols approved by the Augusta University Animal Care and Use Committee. An MI model was generated as previously described. Briefly, MI was induced in 8-week-old C57/B6 mice weighing 22–25 g (The Jackson Laboratory), which were anesthetized with 2% isoflurane (isoflurane USP, Henry Schien). The mice were incubated with a 24-gauge tube and ventilated using a Harvard Rodent Ventilator (MiniVent Type 845, Holliston, MA, United States). Before incision, mice were treated with buprenorphine SR (1.0 mg/kg). The left anterior descending (LAD) coronary artery was permanently ligated with a prolene #8-0 suture. Ten minutes after LAD ligation, 20 μL EVs (particle concentration: 1 × 10^12^/mL) from different groups of C-MSCs or 5 × 10^5^ C-MSCs suspended in 20 μL PBS were injected into the myocardium bordering infarction zone. Before transplantation, C-MSCs were labeled with LuminiCell Tracker 540 (Millipore) according to the manufacturer’s protocol. The same volume of PBS was injected in the control group.

### Echocardiography

Echocardiography was performed in mice anesthetized mildly with inhaled isoflurane (0.5%) using a Vevo 2100 imaging system (VisualSonics Inc.). Hearts were imaged in 2D parasternal short-axis view at the level of the mid-papillary muscle. The M-mode images were used to measure left ventricular end diastolic diameter (LVDd) and left ventricular end systolic diameter (LVDs). Left ventricular ejection fraction (EF) and left ventricular fractional shortening (FS) were analyzed using LV trace for three consecutive cardiac cycles.

### Immunostaining

Hearts were fixed with 4% PFA for 1 h at room temperature and then immersed in 30% sucrose overnight at 4°C. At day 2, hearts were cryopreserved in an optical cutting temperature (OCT) compound (Tissue Tek) at -80°C. Hearts were sliced into 5-μm-thick frozen sections and incubated with primary antibodies including α-sarcomeric actinin (A7811, Sigma, 1:200), ki67 (ab16667, abcam, 1:500), and SMA (ab5694, abcam, 1:300). Slides were incubated with anti-rabbit/mouse secondary antibodies conjugated to Alexa Fluor 594, Alexa Fluor 647, or Alexa Fluor 488 (Life Technologies). Images were taken using a confocal microscope (FV1000, Olympus, Japan). Ki67-positive CMs were analyzed in 22 animals (*n* = 5 in PBS and C-MSC^Notch1 KO^ groups; *n* = 6 in EV-C-MSC and EV-C-MSC^N1ICD^ groups) at 7 days after MI and 12 animals (*n* = 3) at 1 month post-MI. The proliferating CMs were blindly counted in 132 sections (six sections cut at 400 μm intervals from apex to base per heart) at 7 days post-MI and 72 sections at 1 month post-MI. Vessel density was assessed in 20 animals (*n* = 5 in each group) 1 month post-MI. The number of vessels was blindly counted in 60 sections (three sections per heart, 15 sections per group) in the infarct and border areas of all mice after staining with an antibody α-SMA using a fluorescence microscope at a magnification of 400. Vascular density was determined by counting α-SMA-positive vascular structures. The number of vessels in each section was averaged and expressed as the number of vessels per field (0.2 mm^2^). For cells, C-MSCs were fixed with 4% PFA and blocked with 10% FBS, followed by incubation of anti-N1ICD antibody (sc-376403, Santa Cruz) and secondary antibody conjugated to Alexa Fluor 594 (Life Technologies).

### TUNEL Staining

Twenty-four hours after LAD ligation, mice were sacrificed, and heart tissue was embedded and sectioned for TUNEL staining. TUNEL staining was performed using a kit (Thermo Fisher Scientific) according to the manufacturer’s instruction. CMs were identified by α-sarcomeric actinin staining. Cells were counterstained with DAPI to visualize nuclei. Total number of α-sarcomeric actinin and TUNEL double-positive cells were determined in five fields (20 ×) from the border area in each heart (*n* = 3). PBS-treated mice served as control groups.

### Trichrome Masson Staining

Hearts were embedded in paraffin and cut into 5-μm-thick sections. Masson trichrome staining was carried out for scar tissue measurement according to the manufacturer’s protocol (HT-15, Sigma). The size of the left ventricle (LV) area and scar area were measured using the ImageJ software. Six sections with 400 μm intervals from apex to basal were analyzed per heart. The fibrosis area was expressed as the ratio of scar area to LV area.

### Western Blotting

Extracellular vesicles or C-MSCs were lyzed with radio immunoprecipitation assay (RIPA) buffer supplemented with Complete Protease Inhibitor Mixture tablets (Roche Diagnostics). The cell lysate was sonicated in ice using a sonication device (Sonic dismembrator Model100, Fisher Scientific). Five micrograms of protein of EVs or 10 μg protein of cells was separated by SDS/PAGE and transferred to PVDF membrane (BioRad). Membranes were incubated with rabbit anti-CD63 antibody (EXOAB-CD63A-1, SBI System Biosciences), mouse anti-Tsg101 antibody (sc-7964, Santa Cruz), mouse anti-calnexin antibody (sc-23954, Santa Cruz), anti-N1ICD antibody (sc-376403, Santa Cruz), or anti-GADPH antibody (sc-32233, Santa Cruz) overnight at 4°C followed by incubation with an anti-mouse or anti-rabbit goat peroxidase conjugated secondary antibody. Immunoreactive bands were visualized by the enhanced chemiluminescence method (Bio-Rad) with a western blotting detection system (Fluorchem E, ProteinSimple, United States).

### Proteomic Profile by Mass Spectrometry

Sample preparation and analyses were performed at the Proteomics and Mass Spectrometry Core Laboratory at Augusta University. Briefly, EV proteins were extracted and solubilized with acid-labile surfactant, followed by trypsin digestion and peptide cleanup using a C18 spin column. Peptide digests were analyzed on an Orbitrap Fusion tribrid mass spectrometer (Thermo Scientific) coupled with an Ultimate 3000 nano-UPLC system (Thermo Scientific). Two microliters of reconstituted peptide was first trapped and washed on a Pepmap100 C18 trap (5 μm, 0.3 × 5 mm) at 20 μL/min using 2% acetonitrile in water (with 0.1% formic acid) for 10 min and then separated on a Pepman 100 RSLC C18 column (2.0 μm, 75 μm × 150 mm) using a gradient of 2–40% acetonitrile with 0.1% formic acid over 40 min at a flow rate of 300 nL/min and a column temperature of 40°C.

Samples were analyzed by data-dependent acquisition in positive mode using an Orbitrap MS analyzer for a precursor scan at 120,000 FWHM from 300 to 1500 *m/z* and an ion-trap MS analyzer for MS/MS scans at top speed mode (3 s cycle time). Collision-induced dissociation (CID) was used as a fragmentation method. Raw data were processed using Proteome Discoverer (v1.4, Thermo Scientific) and submitted for SequestHT search against the Uniprot human database. The fixed value PSM validator algorithm was used for peptide spectrum matching validation. SequestHT search parameters were 10 ppm precursor and 0.6 Da product ion tolerance, with dynamic carbidomethylation (+57.021 Da). To compare the relative expression difference across different sample groups, the number of PSM was used for each sample as an expression of its relative abundance. The number of PSM was normalized by the sum of PSM in each sample (*n* = 3), which represented the overall sample loading amount on the column. Go terms for enriched protein were analyzed using QuickGO (Binns et al., 2009).

### Statistical Analysis

Data were expressed as mean ± SD. After a test for normality, statistical analysis of differences among different groups was compared by ANOVA with Bonferroni’s correction for multiple comparisons. Differences were considered statistically significant at *P* < *0.05*. Statistical analyses were performed using Graphpad Prism 6.0 (Chicago, IL, United States).

## Results

### *In vivo* Effect of C-MSC Transplantation on Ischemic Injury in an MI Model

**FIGURE 1:**
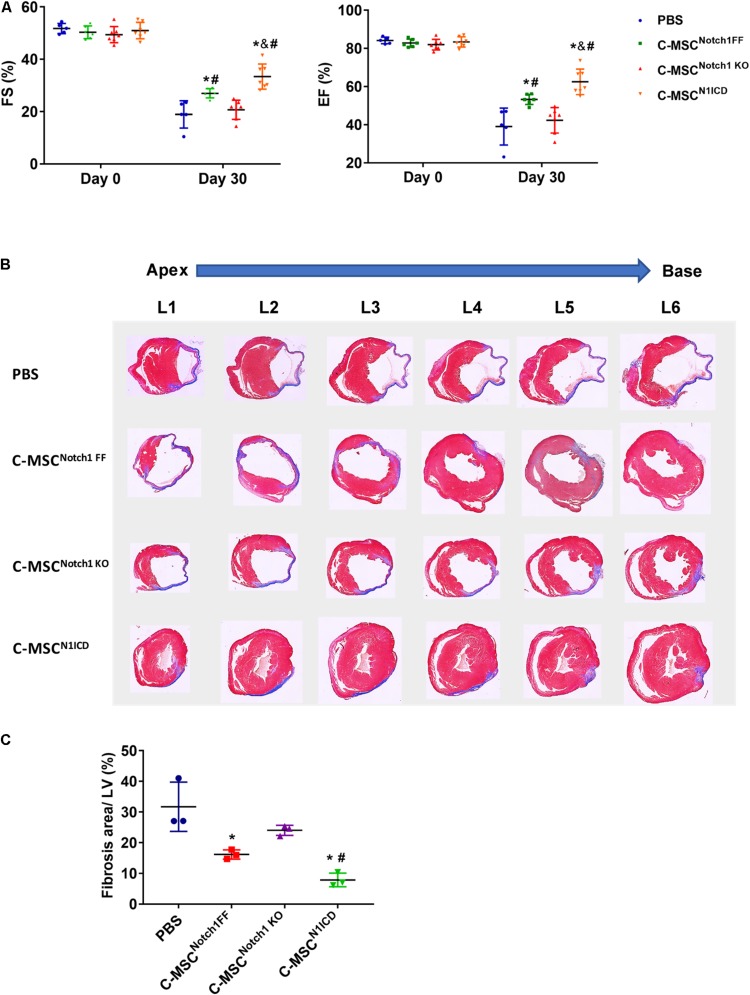
Notch1 intracellular domain (N1ICD)-overexpressing cardiac mesenchymal stem cells (C-MSC) (C-MSC^N1ICD^) transplantation improved cardiac function and attenuated cardiac fibrosis after myocardial infarction (MI). **(A)** Intramyocardium transplantation of both C-MSCs^Notch1 FF^ and C-MSCs^N1ICD^ improved fractional shortening (FS) and ejection fraction (EF) compared with PBS-treated mice (*P* < 0.001). *n* = 5 in PBS, *n* = 7 C-MSC^Notch1 KO^ and Exo-C-MSC^N1ICD^ groups; *n* = 6 in C-MSC^Notch1 FF^ group. **(B)** Representative Masson trichrome-stained sections of hearts from mice treated with C-MSC^Notch1 FF^, C-MSC^Notch1 KO^, and C-MSC^N1ICD^ 1 month after MI. **(C)** Quantitative analysis of fibrosis size from different treated mice 1 month after MI C-MSCs^N1ICD^ significantly decreased fibrosis size compared with other groups, respectively (*n* = 3). * vs. PBS group, # vs. C-MSC^Notch1 KO^ group, & vs. C-MSC^Notch1 FF^, *P* < 0.01.

First, we verified Notch1 deletion or N1ICD overexpression in C-MSCs by western blot (Supplementary Figure S1A). The transfection efficiency of Ad-GFP-N1ICD was more than 95% as visualized by GFP fluorescence (Supplementary Figure S1B). By immunostaining, N1ICD expression was localized in C-MSC nuclei (Supplementary Figure S1C). Next we determined the role of Notch1 in the effectiveness of C-MSCs on cardiac injury. Transplantation of C-MSCs^Notch1 FF^ improved cardiac function compared with the PBS control group (EF: 53.21 ± 2.68% vs. 39.04 ± 9.69%; FS: 27.00 ± 1.78% vs. 18.92 ± 5.21%, *P* < *0.05*) (Figure 1A) and decreased infarct size 1 month post-MI (Figure 1B). Transplantation of Notch1 knockout C-MSCs did not have any effect on cardiac function (EF: 42.32 ± 6.67%; FS: 20.68 ± 3.66%, *P* > *0.05*) and scar size (Figure 1). On the other hand, C-MSCs^N1ICD^ had a significant protective effect on EF and FS (EF: 62.47 ± 6.70%; FS: 33.37 ± 4.79%, *P* < *0.05*) and fibrosis (Figure 1) compared to all other groups (*P* < 0.01). For fibrosis assessment, *n* = 3 was used in each group. Besides their beneficial effect on cardiac function, these cells were colocalized with smooth muscle cells and blood vessels (Supplementary Figure S2). A profound effect of N1ICD-overexpressing C-MSCs was observed on cardiac function and vascularization.

### Characterization of EVs From C-MSCs

**FIGURE 2:**
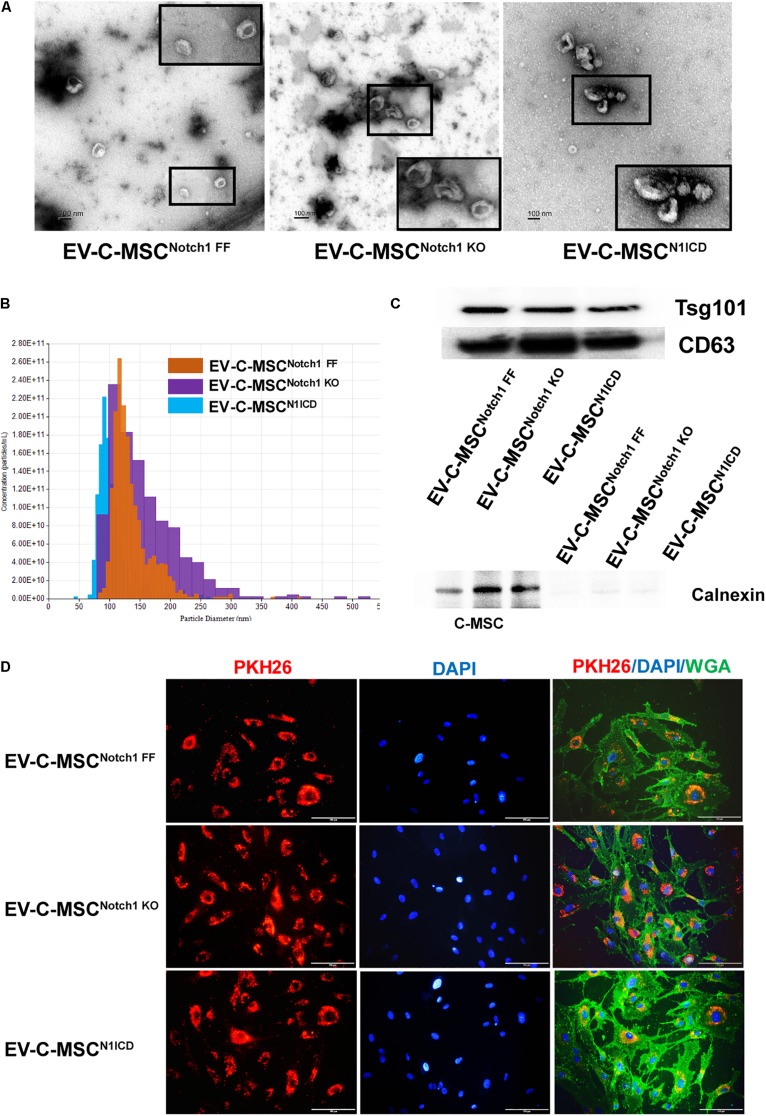
Characterization and internalization of extracellular vesicles from C-MSCs. **(A)** Extracellular vesicles (EVs) secretion from the different C-MSCs as imaged by transmission electron microscopy (TEM). Inset shows higher magnification of EVs. Bar = 100 nm. **(B)** Representative graph of size distribution of EVs from C-MSCs^Notch1 FF^, C-MSCs^Notch1 KO^, and C-MSCs^N1ICD^ detected by tunable resistive pulse sensing (TRPS). **(C)** Representative images of western blot showed that EVs from C-MSCs^Notch1 FF^, C-MSCs^Notch1 KO^, and C-MSCs^N1ICD^ were enriched in EV-specific marker CD63 and Tsg101. Negative marker calnexin was not expressed in EVs. **(D)** EV internalization in cardiac microvascular endothelial cells (CMVECs). PKH26-labeled EVs (red) from different groups of C-MSCs were observed inside the CMVECs (green, WGA), mostly located at the perinuclear region. Bar indicates 100 μm.

We isolated and characterized EVs from different C-MSCs. The appearance and size of EV from C-MSCs^Notch1 FF^, C-MSCs^Notch1 KO^, and C-MSCs^N1ICD^ under TEM and TRPS were similar (Figures 2A,B). EVs from all C-MSCs expressed exosome specific markers Tsg101 and CD63, while they did not express calnexin (Figure 2C).

### Internalization of EVs From C-MSCs by ECs

The therapeutic efficacy of EVs depends on their internalization by recipient cells where they release their contents (Camussi et al., 2010; Feng et al., 2014). Upon incubation with CMVEC, these were internalized by CMVEC and observed in the perinuclear region (Figure 2D). However, in order to rule out the possibility of excess dye remaining, we used an equal concentration of dye in equal volume as used in EV labeling and incubated it with ECs after filtration. After filtration, no red color was observed in the fluid after filtration, suggesting no retention of the dye (Supplementary Figure S3A). In addition, no red fluorescence signal was detected when incubated with EC (Supplementary Figure S3B).

### Proteomic Profile in EVs Derived From N1ICD-Overexpressing C-MSCs

**FIGURE 3:**
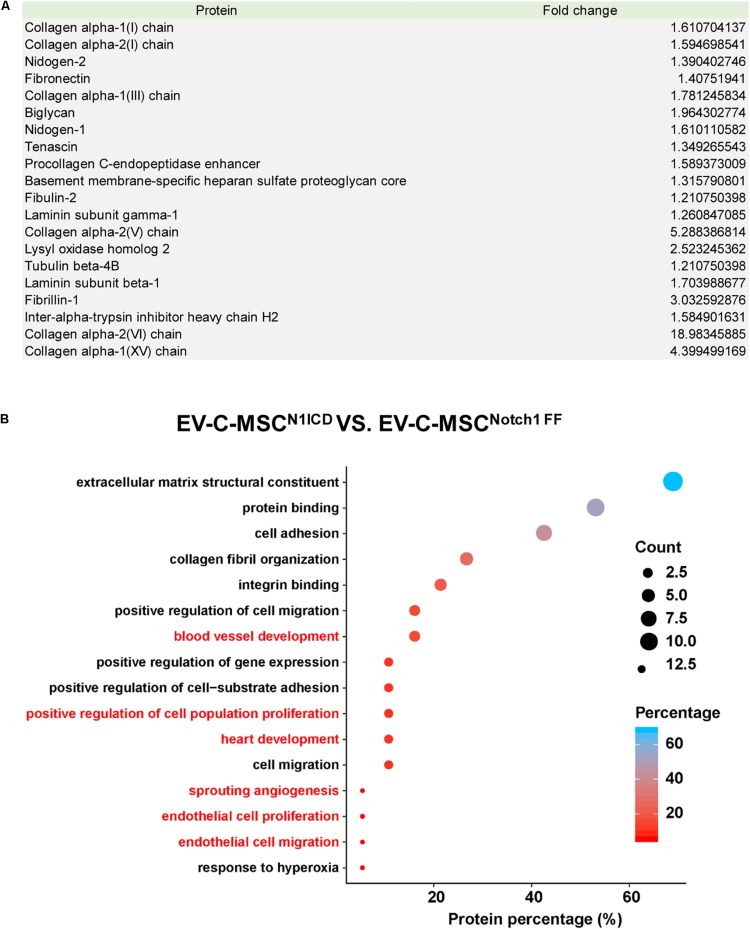
Proteomic profile in EVs derived from N1ICD-overexpressing C-MSCs. **(A)** List of enriched proteins in EV-C-MSCs^N1ICD^ relative to EV-C-MSCs^Notch1 FF^. **(B)** GO protein annotation and pathway enrichment analysis. **A**, Pathway enrichment analysis on differentially expressed proteins in EVs. As expected, many terms referring to biological processes related to various functions of C-MSCs, including extracellular matrix, collagen, proliferation, apoptosis, cell cycle, differentiation, migration, heart development, and angiogenesis were pointed out.

Next, we explored the protein cargo in EVs derived from N1ICD-overexpressing C-MSCs using mass spectrometry. Figure 3A shows a list of enriched proteins in EV-C-MSCs^N1ICD^ relative to EV-C-MSC^Notch1 FF^. Differentially expressed proteins of EVs were annotated on the basis of the GO terms. Considering the EV data set, we found GO terms significantly enriched in the “biological process” category. Proteomics profiling identified several proteins, such as Lysyl oxidase homolog-2 and biglycan, as highly enriched proteins in EV-C-MSCs^N1ICD^ vs. EV-C-MSCs^Notch1 FF^. The enriched proteins in EVs derived from N1ICD-overexpressing C-MSCs were related to blood vessel development, cell proliferation, angiogenesis, EC proliferation and migration, heart development, and response to hypoxia (Figure 3B). Proteomic raw data are available in ProteomeXchange with identifier PXD016578.

### Effect of EVs From N1ICD-Overexpressing C-MSCs on Apoptosis and Tube Formation *in vitro*

**FIGURE 4:**
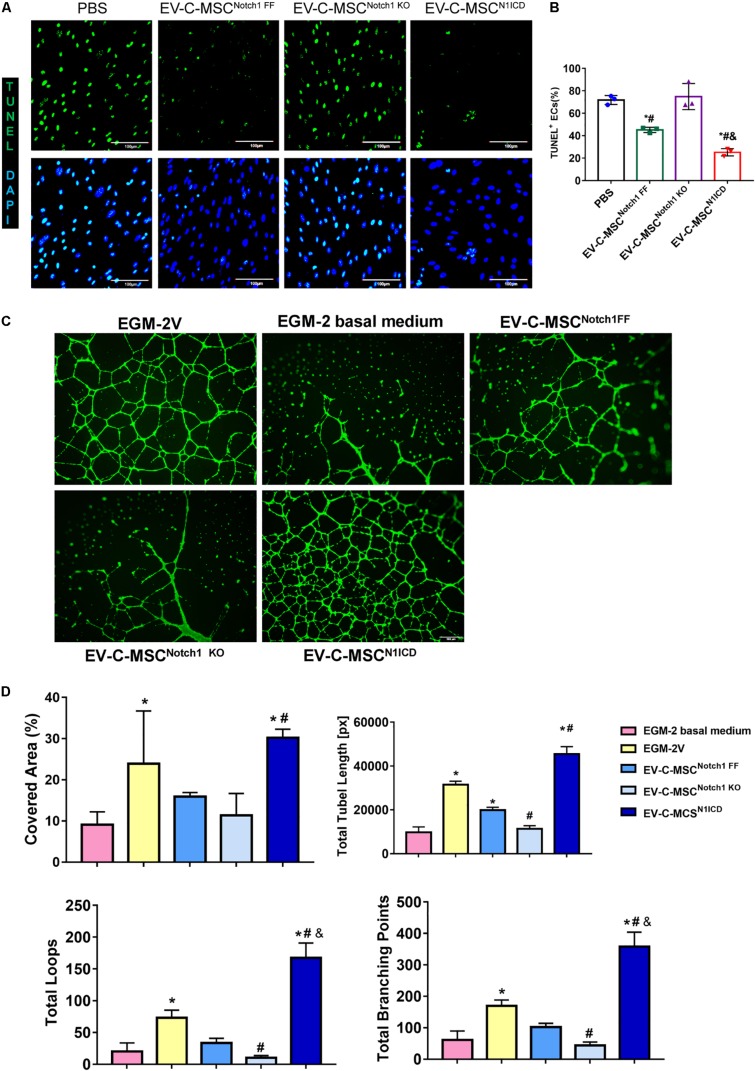
*In vitro* cardioprotective effects of EV-C-MSCs^N1ICD^. **(A)** Representative images of TUNEL staining in mouse ECs which were pretreated with EVs from C-MSCs^Notch1 FF^, C-MSCs^Notch1 KO^, and C-MSCs^N1ICD^ (1 μg/well, 12-well plate) for 24 h and then were subjected to 8 h H_2_O_2_ (300 μM) treatment. Bar = 100 μm. **(B)** Quantitative analysis for TUNEL staining in mouse ECs. * vs. PBS group, # vs. EV-C-MSC^Notch1 KO^ group, & vs. EV-C-MSC^Notch1 FF^ group, *P* < 0.05. **(C)** Representative images of tube formation in human aortic endothelial cells (HAECs) with EV treatment from C-MSCs^Notch1 FF^, C-MSCs^Notch1 KO^, and C-MSCs^N1ICD^ (1 μg/well, 24-well plate). EGM-2V medium and EGM-2V basal medium (without VEGF) served as controls. HAECs were labeled with Calcein AM (Green). Bar = 500 μm. **(D)** Quantitative evaluation for tube formation assay. Covered area, total tube length, total branching points, and total loops were analyzed from three biological repeated experiments. Bar indicates 500 μm. * vs. EGM-2 basal medium group, # vs. EV-C-MSC^Notch1 FF^ group, & vs. EGM-2V group, *P* < 0. 05.

We wanted to determine whether EVs derived from N1ICD-overexpressing C-MSCs had an effect on apoptosis and tube formation. We successfully isolated mouse aortic EC which expressed CD31 (Supplementary Figure S4). Under oxidant stress, pretreatment of EC with EVs from C-MSCs attenuated apoptosis as detected by TUNEL staining (Figures 4A,B). Interestingly, EVs from Notch1 knockout C-MSCs abrogated such effects. Moreover, apoptosis was significantly lower in EV treatment from C-MSCs^N1ICD^ than control or Notch1 knockout C-MSCs (Figures 4A,B). Given the role of Notch1 signaling activation in postnatal angiogenesis, we also determined the effects of EV on tube formation. Using the HAECs’ tube formation assay, we found that EV-C-MSCs^N1ICD^ dramatically promoted tube formation *in vitro* compared to PBS control or EVs from wild-type C-MSCs or Notch1 knockout C-MSCs (Figure 4C). Covered area, total tube length, total branching points, and total loops were all significantly increased with treatment of EV-C-MSCs^N1ICD^ when compared to EVs from other C-MSCs and control groups (Figure 4D).

### *In vivo* Effect of EVs Derived From C-MSCs on Ischemic Injury in Mouse MI Model

**FIGURE 5:**
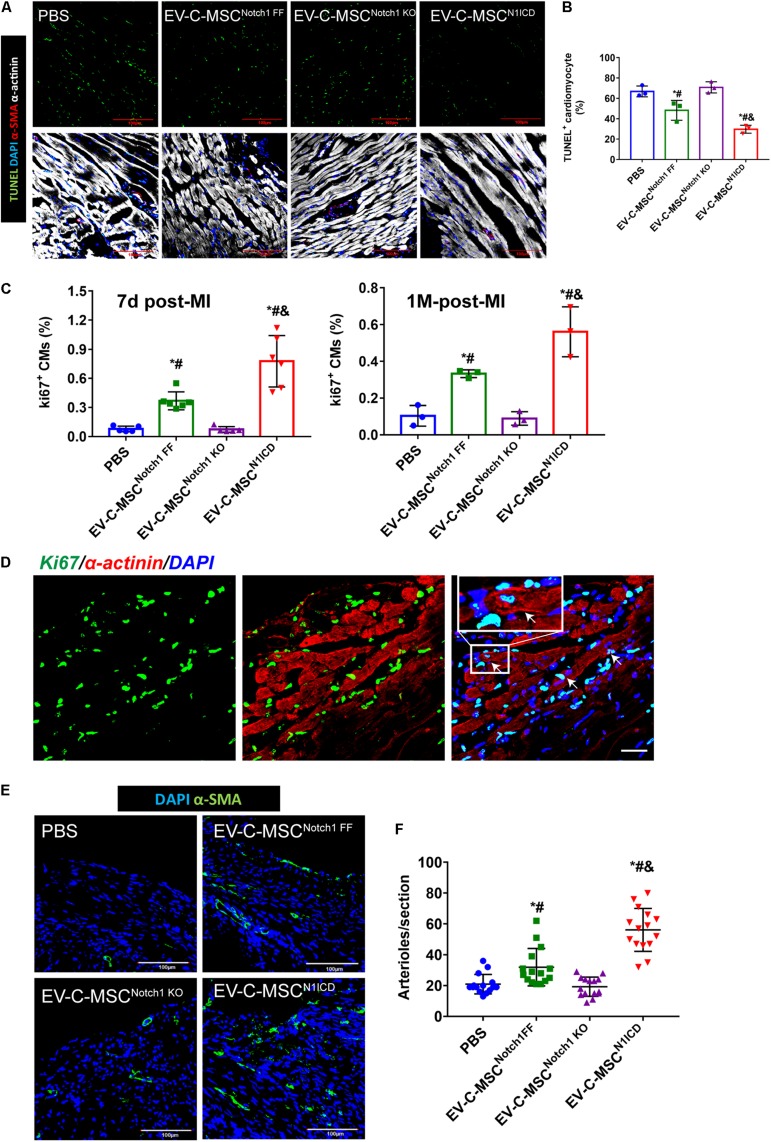
*In vivo* cardioprotective effects of EV-C-MSCs^N1ICD^. **(A)** Representative images of TUNEL staining in infarcted mouse hearts with EV treatment from C-MSCs^Notch1 FF^, C-MSCs^Notch1 KO^, and C-MSCs^N1ICD^ 24 h after MI. **(B)** Quantitative analysis for TUNEL staining in infarcted mouse hearts. * vs. PBS group, # vs. EV-C-MSC^Notch1 KO^ group, & vs. EV-C-MSC^Notch1 FF^ group, *P* < 0.05. **(C)** Quantitative analysis for ki67-positive cardiomyocytes (CMs) in mice with EV treatment from C-MSCs^Notch1 FF^, C-MSCs^Notch1 KO^, and C-MSCs^N1ICD^ at 7 days and 1 month after MI. * vs. PBS group, # vs. EV-C-MSC^Notch1 KO^ group, & vs. EV-C-MSC^Notch1 FF^ group, *P* < 0.001. *n* = 5 in PBS and C-MSC^Notch1 KO^ groups; *n* = 6 in EV-C-MSC and EV-C-MSC^N1ICD^ groups at 7 days after MI; and *n* = 3 at 1 month post-MI. **(D)** Representative image of ki67-positive CMs (α-actinin-positive) in EV-C-MSC^N1ICD^-treated mouse 7 days after MI. Inset shows higher magnification, and white arrows indicate ki67-positive CMs. Bar = 50 μm. **(E)** Representative images of arteriole density in peri-infarct area from mice 1 month after MI. Arterioles were identified by α-SMA positive-staining (green) in the vessles. Bar = 100 μm. **(F)** Quantitative analysis of arteriole density from MI mice with different EV treatment. Arteriole density was markedly increased in EV-C-MSC^N1ICD^-treated hearts in the peri-infarct area compared with other treatments respectively. * vs. PBS group, # vs. EV-C-MSC^Notch1 KO^ group, & vs. EV-C-MSC^Notch1 FF^ group, *P* < 0.05. *n* = 15 sections from five mice in each group.

**FIGURE 6:**
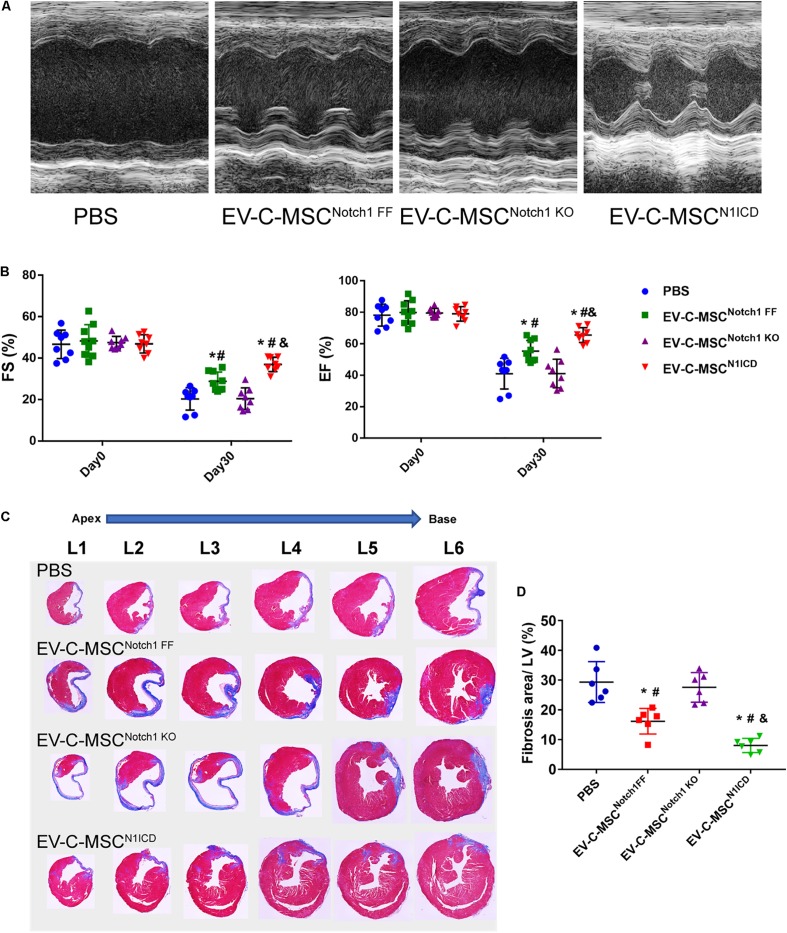
EV-C-MSCs^N1ICD^ improved cardiac function and attenuated cardiac fibrosis after MI. **(A)** Representative M-mode echocardiography images from different EVs treated. **(B)** Intramyocardium injection of both EV-C-MSCs^Notch1 FF^ and EV-C-MSCs^N1ICD^ improved fraction shortening (FS) and ejection fraction (EF) compared with PBS-treated mice (*P* < 0.001). *n* = 8 in PBS, EV-C-MSC^Notch1 KO^, and EV-C-MSC^N1ICD^ groups; *n* = 9 in EV-C-MSC^Notch1 FF^ group. **(C)** Representative Masson trichrome-stained sections of hearts from mice treated with EV-C-MSC^Notch1 FF^, EV-C-MSC^Notch1 KO^, and EV-C-MSC^N1ICD^ 1 month after MI. **(D)** Quantitative analysis of fibrosis size from different treated mice 1 month after MI. EV-C-MSCs^N1ICD^ significantly decreased fibrosis size compared with other groups, respectively. * vs. PBS group, # vs. EV-C-MSC^Notch1 KO^ group, & vs. EV-C-MSC^Notch1 FF^ group, *P* < 0.01. *n* = 6.

Next we investigated whether EVs derived from N1ICD-overexpressing C-MSCs prevent CM apoptosis, stimulate CM proliferation, and promote neovascularization after MI. Twenty-four hours after MI, we observed both that EV-C-MSCs^Notch1 FF^ and EV-C-MSCs^N1ICD^ decreased CM apoptosis compared with the PBS control group (Figures 5A,B). EV-C-MSCs^N1ICD^ further increased CM proliferation at a higher rate than with EV-C-MSCs^Notch1 FF^ (Figure 5C) at both 7 days and 1 month post-MI. Figure 5D shows representative images of Ki67- and α-actinin-positive CMs in the peri-infarct region of EV-C-MSC^N1ICD^-treated mouse hearts 7 days post-MI. In agreement with our *in vitro data*, the vessel density as identified by α-SMA staining and tube-like structures (Figures 5E,F) in the infarcted region was also increased by treatment with both EV-C-MSCs^Notch1 FF^ and EV-C-MSCs^N1ICD^ but significantly higher in the EV-C-MSC^N1ICD^ group than all other groups (Figures 5E,F). In line with a previous study (Ju et al., 2018), EVs from C-MSCs had, in general, a significant effect on cardiac function compared with PBS control (EF 55.30 ± 6.83% vs. 40.98 ± 9.77%; FS: 28.74 ± 4.57% vs. 20.31 ± 5.38%, *P* < *0.05*.) (Figures 6A,B). However, EV-C-MSCs^N1ICD^ had a profound effect on functional parameters (EF: 65.54 ± 4.77% vs. 55.30 ± 6.83%; FS: 35.84 ± 3.54 vs. 28.74 ± 4.57%, *P* < *0.05*.) (Figures 6A,B). EVs from Notch1 knockout C-MSCs had the opposite effect on cardiac function and reversed the beneficial effect of EV-C-MSCs^N1ICD^ (Figures 6A,B). Histological evidence of a larger amount of collagenous mass by the latter treatment further supports the negative role of EVs from Notch1 knockout C-MSCs (*n* = 6) (Figures 6C,D).

## Discussion

In the adult heart, C-MSCs participate in stromal cardiac tissue renewal by differentiating into SMCs and ECs and releasing a variety of paracrine factors responsible for trophic, angiogenic, and anti-inflammatory effects (Martini et al., 2019). Here we report that transplantation of C-MSCs after N1ICD overexpression has a superior and significant effect on cardiac function improvement and attenuation of cardiac fibrosis compared with C-MSCs. Second, EVs secreted by these modified C-MSCs also played a significant role in cardiac rejuvenation and healing compared to EVs from simple C-MSCs. In contrast, Notch1 deletion resulted in loss of regenerative capabilities of C-MSCs and their EVs as well.

Proangiogenic therapy appeared to be a promising strategy for MI. Neovasculogenesis has the potential to salvage ischemic myocardium at early stages post-MI. Notch1 signaling plays a critical role in postnatal angiogenesis including cardiac angiogenesis during ischemia (Yoshida et al., 2014; Zhou et al., 2018), thus enhancing survival of cardiac cells. Notch1 activation promotes VSMC differentiation of CDC through an RBPJ-dependent signaling pathway (Chen et al., 2012). Consistent with these findings, we discovered that N1ICD-overexpressing C-MSCs differentiated into VSMCs in the infarcted heart after transplantation and led to significant functional improvement and attenuation of fibrosis compared to C-MSCs. Despite low expression of N1ICD, C-MSC transplantation had some therapeutic effect (Moore et al., 2017). It appears that Notch1 activation boosts the function of C-MSCs in the regenerative process. However, Notch1 is altered in aging (Rizzo et al., 2018), which also compromises the function of C-MSCs (Martini et al., 2019). How Notch signaling is affected in the aging heart has not been extensively investigated yet. Cardiac progenitor cells (CPCs) lose their reparative potential as they age (Trac et al., 2019). Spherical aggregation rescued the reparative potential of CPCs from older donors due to increased Notch1 signaling (Trac et al., 2019). Our study clearly demonstrates the importance of Notch1 signaling in C-MSC activation. Although the molecular mechanisms of action by Notch1 in aged C-MSCs remain to be characterized, our study emphasizes its importance not only in MI but also for aging heart diseases.

Cardiac mesenchymal stem cells have the potential to differentiate into cardiac lineage cells in the ischemic myocardium, but significant improvement in cardiac function does not correspond to regeneration against scar area by stem cells (Mirotsou et al., 2011). Transdifferentiation and paracrine signaling are suggested to underlie their cardiac reparative effects. More recently, the EVs secreted by stem cells have drawn more attention as final effectors of protection against ischemic injury. Majority of recent studies support the notion that MSCs mediate their effect by paracrine factors (Timmers et al., 2011; Ju et al., 2018). This paracrine signaling is now believed to occur through the release of small vesicles. The therapeutic efficacy of EVs is dependent not only on the synergy of a select permutation of individual EV components but also on the amount of EVs, their internalization by recipient cells, and release of their contents (Camussi et al., 2010; Feng et al., 2014). Manipulation of Notch activity for increased cellular survival and proliferation has recently been promoted as a potential approach for regenerative medicine (Androutsellis-Theotokis et al., 2006). Our data support the notion that EVs from Notch1 engineered C-MSCs have a superior and significant effects on angiogenesis and myocyte proliferation in the ischemic heart following coronary artery ligation. EVs secreted by C-MSCs with Notch1 deletion were not effective in preventing apoptosis and myocyte proliferation and angiogenesis. It appears from the proteomic profile that EVs from N1ICD-overexpressing C-MSCs are enriched with bioactive factors, potent pro-repair proteins responsible for cell migration and proliferation and CM protection. For example, biglycan, one of the enriched proteins in EVs from N1ICD-overexpressing C-MSCs, has been demonstrated as an angiogenic factor (Yamamoto et al., 2012; Xing et al., 2015; Myren et al., 2016). Biglycan enhanced promoter activity of hypoxia-inducible factor-1α (HIF-1α), resulting in increased HIF-1α mRNA levels, as well as augmented HIF-1 activity, leading to increased VEGF expression (Hu et al., 2016). Lysyl oxidase-like protein-2 (LOXL-2), a highly enriched protein, was also related to regulation of sprouting angiogenesis and played an essential role in developmental angiogenesis (Zaffryar-Eilot et al., 2013). It is expressed in neovessels as a hypoxia target and accumulated in the endothelial extracellular matrix (ECM) (Bignon et al., 2011). A gain-and-loss-of-function experiment demonstrated that LOXL-2 overexpression increased capillary formation and LOXL-2 knockdown dramatically reduced EC migration and proliferation, resulting in decreased tubulogenesis (Bignon et al., 2011; de Jong et al., 2019). In addition, biglycan overexpression in transgenic mice has been shown to induce cardioprotective genes [nitric oxide (NO) synthases] in the heart (Bereczki et al., 2007). Biglycan protected CMs against hypoxia/reoxygenation injury in an NO-dependent mechanism (Csont et al., 2010). Consistent with these studies, EVs from N1ICD-overexpressing C-MSCs prevented apoptosis of ECs and CMs exposed to oxidative stress and ischemic injury and promoted cardiac angiogenesis. It is likely that biglycan and LOXL-2 contributed to such protective effects. Sprouting angiogenesis is related to extensive ECM remodeling (Neve et al., 2014; Crosby and Zoldan, 2019). Multiple levels of cell–ECM interactions are potentially involved in capillary formation (Edgar et al., 2014; Rauff et al., 2019). We noticed that some laminin subunits and collagen alpha chains were upregulated in EVs from C-MSCs^N1ICD^. Thus, the molecular mechanisms of action by these proteins in EVs remain to be determined.

## Conclusion

Notch signaling is important in cardiac repair following myocardial injury. In this study, we have investigated the role of EVs from Notch1 engineered C-MSCs in angiogenesis and CM proliferation in ischemic myocardium. EV-C-MSCs^N1ICD^ were very effective in improving cardiac function and decreasing fibrosis. Notch1 signaling is a strong stimulus for cardiac regeneration by C-MSCs. EVs secreted by Notch1-overexpressing C-MSCs were highly effective in preventing cell death, promoting angiogenesis and CM proliferation, and restoring cardiac function post-MI. In conclusion, proangiogenic factors from EVs of N1ICD-overexpressing C-MSCs might be a novel strategy for boosting angiogenesis in ischemic hearts.

## Data Availability Statement

The proteomic raw data generated in this study has been deposited in ProteomeXchange with identifier PXD016578.

## Ethics Statement

All study protocols were approved by the Institutional Animal Care and Use Committee and carried out consistent with the recommendations of the American Veterinary Medical Association guidelines.

## Author Contributions

WX participated in experimental design, acquisition, and analysis of experimental data, and drafted the manuscript. MK helped in experimental design, manuscript writing, and proofreading. MA conceived the idea, helped in experimental design, finalized the manuscript, and financially supported the study through NIH funding.

## Conflict of Interest

The authors declare that the research was conducted in the absence of any commercial or financial relationships that could be construed as a potential conflict of interest.
