# Supplementary material for: Extracellular Vesicles From Notch Activated Cardiac Mesenchymal Stem Cells Promote Myocyte Proliferation and Neovasculogenesis
Source: Front Cell Dev Biol. 2020 Feb 21;8:11. doi: 10.3389/fcell.2020.00011 (PMC7047205; doi:10.3389/fcell.2020.00011)
Supplement: Supplementary file 1 [file Data_Sheet_1.pdf]

Supplementary Material

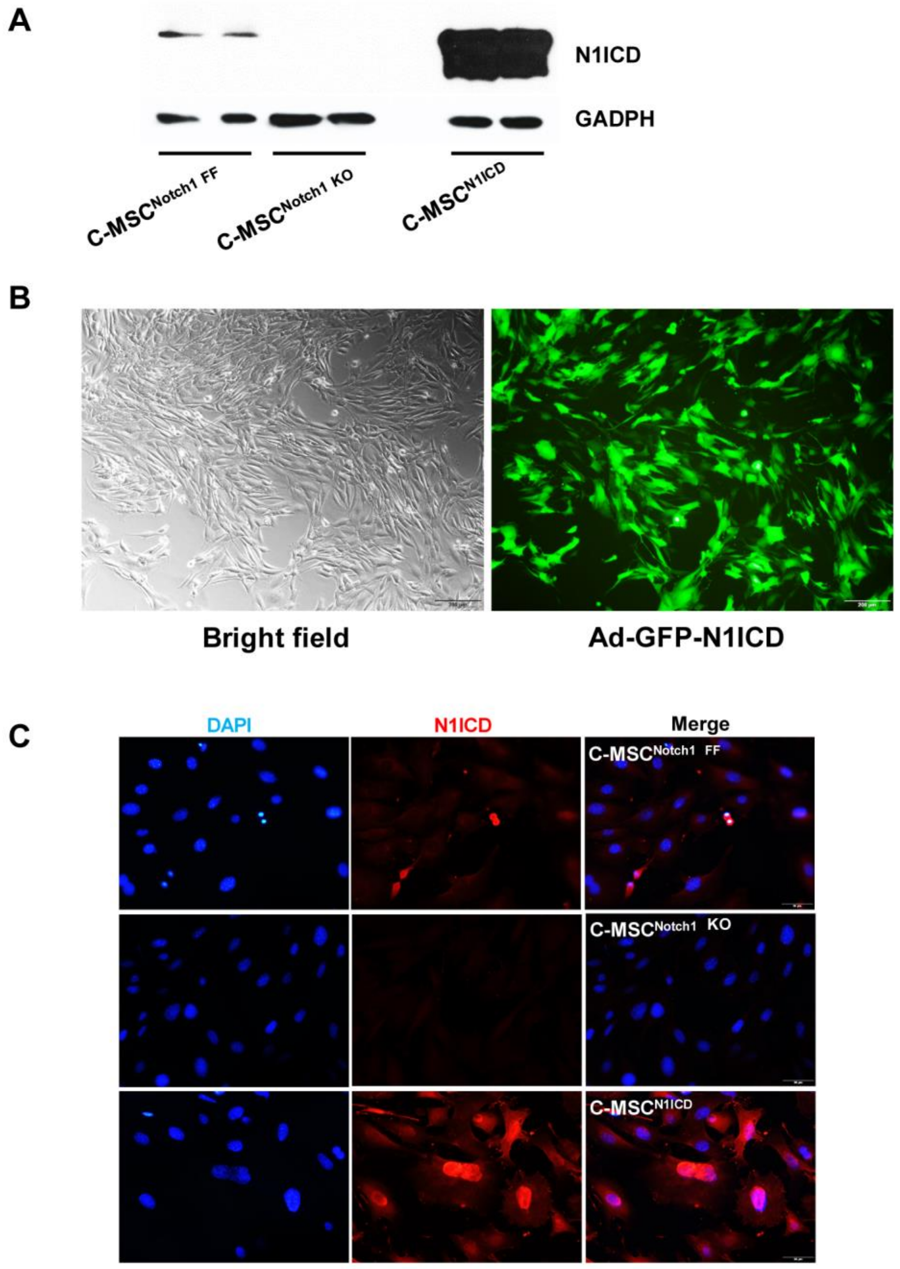

Supplemental Fig.S1 Overexpression of Notch1 intracellular domain (N1ICD) in Cardiac Mesenchymal stem cells (C-MSC). (A) Representative western blot images of N1ICD expression in C-MSC from Notch1flox mice, Notch1 knock out C-MSC and N1ICD overexpressing C-MSC. (B) Ad-green fluorescence protein (GFP)-N1ICD was infected into C-MSC. Transfection efficiency was monitored by fluorescence microscopy. Bar = 200 μm. (C) Representative immunofluorescence images of N1ICD expression in C-MSC from Notch1 flox mice, Notch1 knock out C-MSC and N1ICD overexpressing C-MSC. Bar = 50 μm.

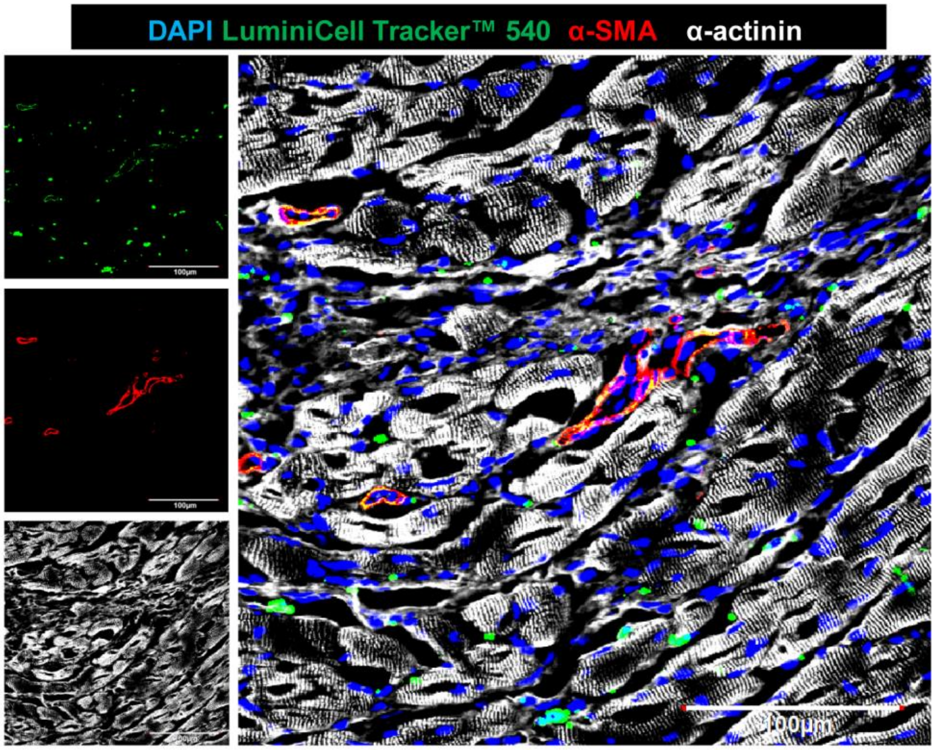

Supplemental Fig.S2 N1ICD overexpressing C-MSC differentiated into smooth muscle cells after in vivo transplantation in infracted mouse heart. Cells were labeled with LuminiCell Tracker™ 540. Co- localization cell tracker and α-SMA staining was observed indicating cells were differentiated into smooth muscle cells and formed vessels. Bar = 100 μm.

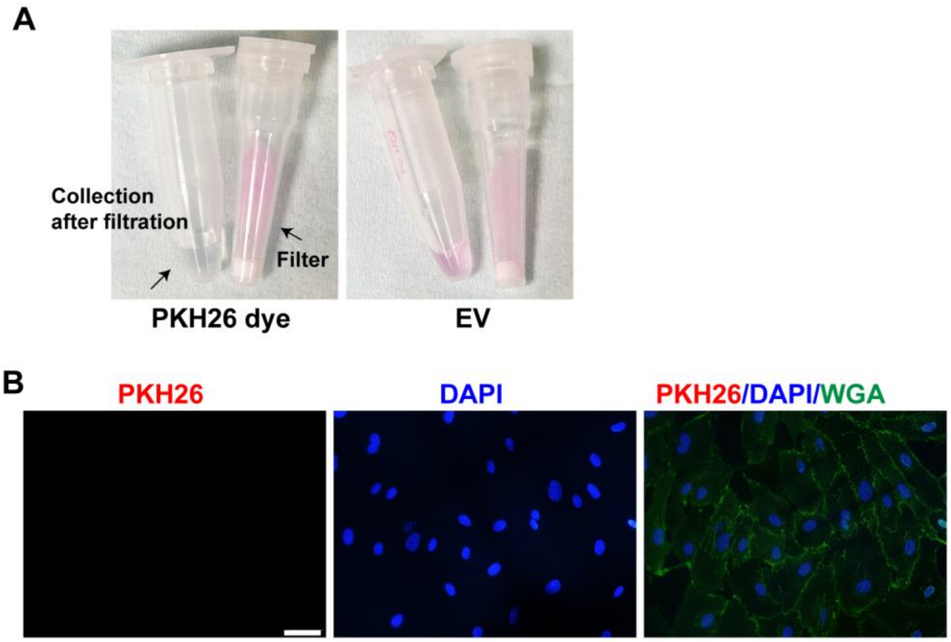

Supplemental Fig.S3 Excess dye removal for EV labeling. (A) Appearance of Filtration fluid of PKH26 labeled EV or PKH26 dye. (B) No PKH26 dye signal was detected in endothelia cells incubated filtration fluid of PKH26 dye. Bar = 50 μm.

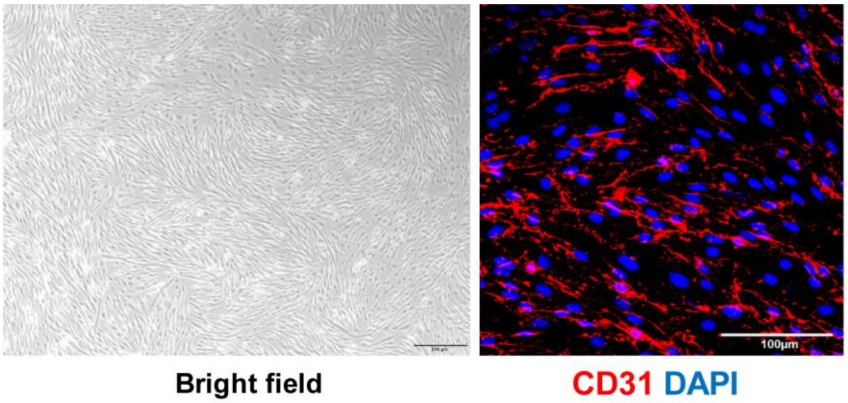

Supplemental Fig.S4 Characterization of mouse aortic endothelia cells. Representative bright filed image of mouse aortic endothelia cells (Bar = 200 μm) and immunofluorescence image of CD31 expression in mouse aortic endothelia cells (Bar = 100 μm).
